# A Systematic Review of Meta-Analyses Comparing Periodized and Non-periodized Exercise Programs: Why We Should Go Back to Original Research

**DOI:** 10.3389/fphys.2019.01023

**Published:** 2019-08-07

**Authors:** José Afonso, Tiago Rocha, Pantelis T. Nikolaidis, Filipe Manuel Clemente, Thomas Rosemann, Beat Knechtle

**Affiliations:** ^1^Faculty of Sport, Centre for Research, Education, Innovation and Intervention in Sport, University of Porto, Porto, Portugal; ^2^Master Science Lab, Vila Nova de Gaia, Portugal; ^3^Exercise Physiology Laboratory, Nikaia, Greece; ^4^School of Sport and Leisure, Polytechnic Institute of Viana Do Castelo, Melgaço, Portugal; ^5^Institute of Primary Care, University of Zurich, Zurich, Switzerland; ^6^Medbase St. Gallen Am Vadianplatz, St. Gallen, Switzerland

**Keywords:** periodization, programming, non-periodized variation, exercise, systematic review

## Abstract

Periodization schedules training periods according to predicted timings of cumulative adaptations and has been at the foundation of exercise prescription. Recently, a selected body of work has highlighted that original research may be providing support for variation, but not for periodized variation. Furthermore, it has been suggested that the timings of expected adaptations have not been tested. However, it is not clear if these problems are present in meta-analyses on the subject, since they might have selected a distinct body or work. Therefore, our goal was to systematically review meta-analyses on exercise periodization, to verify whether the included periodized programs have been contrasted to two types of non-periodized programs (i.e., constant or varied) and also if the predictions concerning cumulative adaptations were tested. *Data sources*: Cochrane, EBSCO (Academic Search Complete, CINAHL Plus, MEDLINE, PsycINFO, SportDISCUS), ISI Web of Knowledge, PEDro, PubMed, Scielo, Scopus. *Study eligibility criteria*: Meta-analyses comparing periodized exercise programs with non-periodized programs. *Participants and interventions*: Humans following any form of training periodization. *Study appraisal and synthesis methods*: A checklist was used to verify whether studies included in the meta-analyses compared periodized to constant or varied, non-periodized programs, as well as whether predictions concerning the timing of adaptations were tested. None of the 21 studies included in the two meta-analyses compared periodized programs with varied, non-periodized programs. The accuracy of the predictions concerning the proposed timings of adaptations was not scrutinized by any of the 21 studies. The studies in question have focused only on strength training, meaning they are limited in scope. The limitations found in these meta-analyses suggest that consultation of original research on the subject is advisable. *Systematic review registration number (PROSPERO)*: CRD42018111338.

## Introduction

Exercise periodization is defined as the systematic planning of training with the aim of achieving the best performances at specific dates, as first discussed in the works of Matveyev (1981). Periodization involves the establishing of fitness phases and timelines (Suchomel et al., 2018), which implies foreseeing—to a certain degree—the timings of adaptations to pre-established load dynamics. In this context, programming is the establishment of the specific training routines to be implemented in each training period or phase (Suchomel et al., 2018). Broadly, periodization establishes the goal or emphasis of the training phases, while programming establishes the means for achieving the proposed parameters. In practice, research on periodization presents a mixture of specific periodized models with specific programming.

Beyond the specificities of the different periodized models, there are common aspects underlying the concept of periodization *per se*: (i) determining relevant dates (e.g., main competitions); (ii) determining fitness phases for each training period; (iii) managing load dynamics with the intent of achieving peak or optimal performance at the previously determined dates (Issurin, 2010; Naclerio Ayllón et al., 2013; Loturco and Nakamura, 2016). The application of systematic variation and the reliance on predictions concerning responses to the load dynamics therefore are transversal and constitute the foundation for periodized programs.

Although periodization implies variation, variation does not imply periodization. Varied programs can be non-periodized whenever that variation is not previously stipulated, rather emerging from an ongoing analysis of the process (i.e., largely unprogrammed variation, or programmed only for the short-term, e.g., a training week). Research has compared periodized programs with constant programs, but comparisons between periodized programs and varied non-periodized programs seems to be lacking (Kiely, 2012; Afonso et al., 2017). Furthermore, different periodized models propose distinct load dynamics and diversified timings for adaptation to occur. The burden of proof requires comparing the expected outcomes with the actual outcomes, but empirical research on the subject seems to have failed to address this issue (Kiely, 2012; Afonso et al., 2017).

Within the context of scientific research, meta-analyses have been considered by some to constitute the top of the pyramid (Wolf, 2015; Cote et al., 2016; Zhi et al., 2017). However, the assumption that meta-analyses provide state-of-the-art rationales for understanding a given phenomenon is not always justified (Tan et al., 2014; Gentil et al., 2017). Indeed, meta-analyses can produce ambiguous results and it is paramount that the quality of such studies is properly scrutinized (Zhi et al., 2017). If not, meta-analysis can amplify research errors (Cote et al., 2016), and consequently may provide a false sense of security in respect to given datasets. In the case of exercise periodization, previous narrative (Kiely, 2012) and systematic reviews (Afonso et al., 2017) have shown two problems in original research (i.e., absence of varied, non-periodized groups; absence of predictions concerning the timing of adaptations). It is not clear, however, if these problems are also present in the existing meta-analyses on the subject, since it is possible that those meta-analyses have selected a different set of original research.

Accordingly, it is our conviction that scrutinizing existing meta-analyses should be a part of the scientific endeavor, even because these types of articles usually produce a great impact on scientific communities. The present work therefore aimed to systematically review meta-analyses of studies on exercise periodization. The first goal was to verify if the included studies have compared periodized programs to both constant and varied, non-periodized programs. The second goal was to assess if the predictions concerning the expected timings of adaptations have been tested.

## Methods

### Protocol and Registration

The full protocol was registered in PROSPERO with the reference CRD42018111338.

### Eligibility Criteria

We included meta-analyses comparing periodized to non-periodized exercise programs that have been published in English, French, Italian, Portuguese, or Spanish. No restrictions were placed with regard to publication date. Both published articles and articles in the press were considered.

Following the Preferred Reporting Items for Systematic Reviews and Meta-Analyses (PRISMA) guidelines, P.I.C.O.S. was established as follows: Participants—humans of any health condition; Interventions—any intervention involving exercise; Comparisons—periodized programs and non-periodized programs (regardless of the physical capacity or skill analyzed); Outcomes—type of non-periodized programs included and presence or absence of predictions concerning the timing of adaptations, i.e., at which points in time certain outcomes are expected to achieve their highest or lowest values (those predictions concerning the outcomes may be qualitative or quantitative); Study design—meta-analyses.

### Information Sources

The following databases were consulted: Cochrane, EBSCO (Academic Search Complete, CINAHL Plus, MEDLINE, PsycINFO; SportDISCUS), ISI Web of Knowledge, PEDro, PubMed, Scielo, and Scopus. As mentioned, no restrictions were imposed in regard to publication date.

### Search

The literature search was conducted in October 2018 and renewed on March 2019. BOOLEAN operators were used for the initial screening. The search terms in the title were associated with (“meta-analysis” OR “meta-analyses”) AND (“periodization” OR “periodized”). The search was repeated with variations of these words (e.g., periodization, periodized).

For PEDro, due to specificities of the database, the words “periodized” and “periodization” were searched for within the title and abstract. The database then presents a number of articles and details whether they are clinical trials, systematic reviews, or meta-analyses.

### Study Selection

After the initial screening, articles were selected if they focused on exercise rather than other protocols (e.g., periodization of nutritional programs). Articles were also selected if they compared periodized programs to non-periodized programs (i.e., meta-analyses comparing different periodized approaches to groups other than non-periodized groups were excluded). Overall, studies were included if they respected PICOS as previously defined.

### Data Collection Process

The main author of this manuscript conducted the first literature review and data extraction. A second author of this manuscript independently repeated the process within the same month and the two processes were compared to ensure that there were no mistakes in the literature search and retrieval process, as well as in the exclusion process. The remaining authors of this work crosschecked the entire process to verify errors or inconsistencies.

Having identified the final list of meta-analyses, each article contained within each meta-analysis was thoroughly reviewed in light of the two research questions: (i) what type of non-periodized programs were used (constant and/or varied); and (ii) were predictions regarding the timing of adaptations tested? Two authors of this manuscript reviewed every original article. After this process, the other two authors repeated the analysis to ensure reliability.

### Data Items

As explained in the rationale, systematic variation and establishment of predictions concerning load dynamics are common to every periodized program, regardless of its specificity. In a sense, these two concepts represent the common ground across all periodized programs. Due to the specific goals of this systematic review, which focus specifically on these two conceptual aspects underlying research on exercise periodization, only two data items were considered. The research articles included in the meta-analyses were analyzed with a 2-fold purpose. First, we assessed the *type of non-periodized program that was used*, which could be constant or varied. As mentioned in the rationale, non-periodized programs do not necessarily have to be constant, and so it was important to understand if non-periodized, varied programs were considered in the analyses.

Secondly, the *qualitative and/or quantitative predictions concerning the timing of adaptations* were assessed. Such predictions could be present (i.e., tested) or absent (i.e., untested). A simple checklist was used to verify these aspects.

### Risk of Bias in Individual Studies

The very nature and goals of this systematic review demanded a simple, but highly specific instrument for evaluating the studies, and we could not find such an instrument in the consulted literature. The construction of the instrument was conceived to answer the two main goals of this work. Risk of bias in individual studies was therefore assessed using a verification list and then converted into a summed score ranging from 0 (unbiased) to 2 (doubly biased). *Type of non-periodized program used:* A score of one was assigned when only constant programs were contrasted with periodized programs, because this fails to compare periodized programs to non-periodized, varied programs. And the two meta-analysis explicitly argued that periodization was superior to non-periodization, but comparing periodized programs to only constant programs supports variation, not periodized variation. *Predictions concerning timing of adaptations*: One point was given when predictions concerning the timing of adaptations were not tested, as these constitute a cornerstone of exercise periodization and one of its main purported advantages.

The binary nature when evaluating each data item allows addressing the conceptual issues tackled by this research, since predictions are either tested or not, and non-periodized, varied programs are either included or not.

### Synthesis of Results

An average risk of bias across studies was attributed to classify each meta-analysis. This considers the fact that non-varied periodized programs might not be contemplated, and that predictions concerning the timing of adaptations may have not been tested. Hence, both meta-analyses can present an average risk of bias between 0 and 2.

## Results

### Study Selection

**Figure 1:**
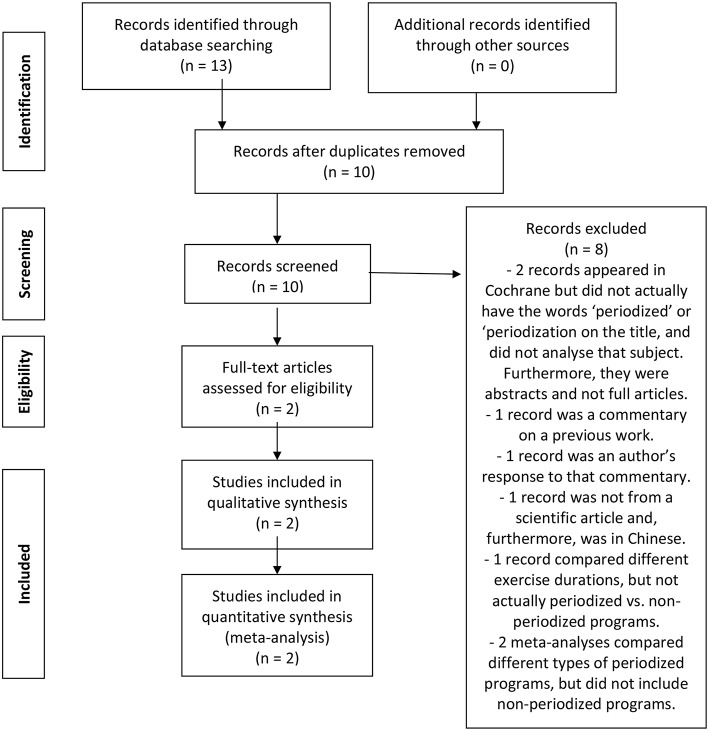
Flow diagram for the search process.

Thirteen studies emerged from the initial search process. Ten studies remained after a manual removal of duplicates. After a screening process, eight studies were excluded: two articles emerged in Cochrane but did not present the search terms in the title, and did not investigate periodization; one article was a commentary on a previous work; one record was an author's response to that commentary; one article was in Chinese and was published in a non-peer-reviewed journal; one article compared different exercise durations, not periodized vs. non-periodized approaches; two meta-analyses compared different periodized approaches and did not include non-periodized programs. As a result, two meta-analyses were assessed for eligibility and deemed appropriate for inclusion in the synthesis. A flow diagram representing this search strategy is presented in Figure 1.

### Study Characteristics

The meta-analysis by Williams et al. (2017) aimed to examine periodized resistance training programs to non-periodized training plans and to determine effects on maximal strength. This analysis included 18 articles. The meta-analysis by Rhea and Alderman (2004) aimed to compare periodized and non-periodized training programs for strength and/or power development, and included 11 articles. Therefore, both meta-analyses have focused on different manifestations of strength training. Periodization of other physical capacities or skills, as well as additional training factors (e.g., technical, tactical, and psychological) were not yet the target of meta-analysis.

### Risk of Bias Within Studies

Each study included in the meta-analyses received a score of 2, meaning they presented maximal risk of bias. This occurred because they did not include varied, non-periodized programs, and also because they did not test predictions concerning the timing of adaptations. However, it should be highlighted that the scope of these meta-analyses happened to be limited to strength training.

### Results of Individual Studies

Results from individual studies within the meta-analyses are presented in Table 1. None of the studies included in these meta-analyses incorporated varied, non-periodized programs, having compared only periodized programs with constant programs. We recognize that periodization and variation are conceptually similar and, although not synonymous, these two concepts are often associated and treated as if inseparable. Furthermore, none of the studies tested qualitative or quantitative predictions concerning the timing of adaptations, namely when their previously stipulated evaluative parameters should peak or fall.

**Table 1:** Results from individual studies included in the two meta-analyses.

**References**	**Included in the meta-analyses of**	**Incorporated non-periodized variation**	**Tested predictions (timing of adaptations)**	**Additional concerns**
Ahmadizad et al. (2014)	Williams et al., 2017	No	No	
Baker et al. (1994)	Rhea and Alderman, 2004; Williams et al., 2017	No	No	
DeBeliso et al. (2005)	Williams et al., 2017	No	No	
Herrick and Stone (1996)	Rhea and Alderman, 2004; Williams et al., 2017	No	No	
Hoffman et al. (2009)	Williams et al., 2017	No	No	
Kraemer et al. (2003)	Williams et al., 2017	No	No	
Kraemer (1997)	Rhea and Alderman, 2004; Williams et al., 2017	No	No	This study analyzed multiple vs. single sets, so should have been excluded.
Kraemer (1997)	Rhea and Alderman, 2004	No	No	This study analyzed multiple vs. single sets, so should have been excluded.
Kraemer et al. (2000)	Rhea and Alderman, 2004; Williams et al., 2017	No	No	This study analyzed multiple vs. single sets, so should have been excluded.
Marx et al. (2001)	Rhea and Alderman, 2004; Williams et al., 2017	No	No	This study analyzed low-volume vs. high volume, so should have been excluded.
McCarthy (1991)	Rhea and Alderman, 2004	No	No	Unpublished work, so should have been excluded.
McGee et al. (1992)	Rhea and Alderman, 2004; Williams et al., 2017	No	No	This study analyzed three different weight-training programs, so should have been excluded.
Monteiro et al. (2009)	Williams et al., 2017	No	No	
Moraes et al. (2013)	Williams et al., 2017	No	No	
O'Bryant et al. (1988)	Williams et al., 2017	No	No	
Pacobahyba et al. (2012)	Williams et al., 2017	No	No	
Schiotz et al. (1998)	Rhea and Alderman, 2004; Williams et al., 2017	No	No	
Souza et al. (2014)	Williams et al., 2017			
Stone et al. (2000)	Rhea and Alderman, 2004; Williams et al., 2017	No	No	This study analyzed three different weight-training programs, so should have been excluded.
Storer et al. (2014)	Williams et al., 2017	No	No	This study analyzed supervised vs. unsupervised training programs, so should have been excluded.
Willoughby (1992)	Rhea and Alderman, 2004	No	No	This study analyzed three different weight-training programs, so should have been excluded.

We identified additional problems with the included meta-analyses. Eight of the 21 studies included did not compare periodized programs with non-periodized programs, and instead compared different concepts altogether (i.e., low-volume vs. high-volume, multiple vs. single sets, supervised vs. unsupervised, different weight training protocols). Additionally, one of the included works was unpublished (McCarthy, 1991) and, therefore, has not been subject to peer-review.

### Synthesis of Results

The studies included in the two meta-analyses did not comply with the two conceptual criteria for researching exercise periodization: neither meta-analysis tested the predictions made by periodized models, nor did either compare periodized variation with non-periodized variation. Moreover, not all of the studies included in these meta-analyses were focused on the theme of periodization, meaning that their presence is highly questionable.

### Risk of Bias Across Studies

Overall, risk of bias across studies was maximal (i.e., 2). The included meta-analyses therefore presented maximal bias and, objectively speaking, did not evaluate the merits of periodized programs, rather only those of varied programs in comparison to constant programs. And even within this more limited scope, several included works did not actually compare varied programs with constant programs, but rather different conceptual subjects such as training volume.

## Discussion

The present systematic review reports on two meta-analyses comparing periodized and non-periodized exercise programs. Meta-analysis are impactful works, but the quality of a meta-analysis is directly dependent on the quality of the studies upon which it is based (Tan et al., 2014; Cote et al., 2016; Gentil et al., 2017). Overall, our research has shown that meta-analyses on exercise periodization do not demonstrate that periodized programs are superior to non-periodized, varied programs. There is also no reliable evidence in these meta-analyses that periodized programs could be used to predict or manage timings of adaptations. Even though the studies included in both meta-analyses have reported baseline and follow-up data, the fact is that each periodized model proposes specific timings for adaptations, and how the results fit within those theoretical timings was not investigated. No predictions can be reasonably put forth outside of any study's length, but predictions for the duration of the study could have been tested.

The study by Rhea and Alderman (2004) included 11 studies that were focused on strength or power development, and all these studies compared periodized programs to constant programs. None of these 11 studies compared periodized programs to non-periodized, varied programs, and so no evidence for the superiority of periodized programs can be derived from this meta-analysis, although most of these articles did show a superiority of periodized programs in relation to constant programs (i.e., the programs were not varied or changed in time). Additionally, not all the articles included in this meta-analysis were randomized, a factor which poses serious questions of validity when interpreting the results (Cote et al., 2016).

The study by Williams et al. (2017) compared periodized and non-periodized resistance training programs with a focus on maximal strength. Non-periodization was equated with a constant program, meaning that none of the 18 studies analyzed compared periodized programs with non-periodized, varied approaches. Moreover, none of these studies attempted to test the predictions of each applied periodized program. This meta-analysis also had more serious shortcomings that cast doubt on the interpretation of its results. One of the included studies, for example, compared a low-volume training protocol (which was not periodized) to a high-volume protocol (which was periodized), and the authors concluded that periodization was superior to non-periodization (Marx et al., 2001). However, because the training volumes were drastically different, it is likely that periodization was not related with the outcomes. Another of the included studies (Storer et al., 2014) compared subjects practicing under supervised vs. non-supervised conditions and concluded that periodization produced superior results. The conclusion should have been that supervised programs produced better results.

Furthermore, these meta-analyses pooled data that should probably not have been pooled. As was shown some articles included in the two meta-analyses researched themes that are clearly not about periodization, but about different issues altogether (e.g., low- vs. high-volume training; supervised vs. unsupervised training). One work was not even published. Our study is thus in agreement with Cote et al. (2016), who stated that articles of insufficient quality should not have their data pooled. Additionally, these meta-analyses are limited to strength training, which is merely one among many factors influencing performance. Since research in periodization is clearly not limited to strength training, meta-analysis can potentially be conducted upon periodization of other training factors.

The current review of meta-analyses should not be interpreted as a criticism of the concept of periodization. We are not stating that non-periodized approaches are superior to periodized approaches, even though there would be precedents for that in literature (Freitas et al., 2019). We are, however, stating that the scientific principle of the burden of proof should be applied (Hamilton and Best, 2011; Koplin and Selgelid, 2015). Therefore, research on periodization should not limit the comparisons of periodized programs to constant programs, and should not be excused of comparing expected timings of adaptations with actual outcomes. We also recognize that there is a considerable body of original research that has not been scrutinized by these meta-analyses. Finally, it is possible that we did not identify meta-analyses in other languages that satisfy the two conditions.

## Perspective

The meta-analyses included in our review did not incorporate studies contrasting periodized approaches to non-periodized, varied approaches, and predictions concerning the timing of adaptations were not tested. Therefore, two concepts that are transversal to all periodized approaches have not been properly evaluated. These specific meta-analyses may have amplified existing research problems, and some of the included studies did not even concern periodization (e.g., supervised vs. unsupervised training). Although absence of proof does not constitute proof of evidence, it does show that research still has a long way to go.

Future research should test the specific predictions made by periodized programs, and investigate if the key to success is variation *per se*, rather than periodized variation (Kiely, 2018). Perhaps there is a continuum from absolutely pre-programmed variation to completely random variation, with success being more likely achieved somewhere in the middle. It is our conviction that most coaches who utilize periodized training programs recognize the need to operate changes depending on how the process is actually evolving and unfolding. Nevertheless, the current understanding of inter- and intra-individual variation in response to training is surprisingly limited, and this should advise us of the limitations of predicting load dynamics in advance.

## Author Contributions

JA conceived the study. JA, TRoc, PN, and FC designed the study, and collected, analyzed the data. JA, TRoc, PN, FC, TRos, and BK interpreted the data and drafted the manuscript. All authors revised the manuscript and approved the final version.

### Conflict of Interest Statement

The authors declare that the research was conducted in the absence of any commercial or financial relationships that could be construed as a potential conflict of interest.
